# Genome-wide association identifies several QTLs controlling cysteine and methionine content in soybean seed including some promising candidate genes

**DOI:** 10.1038/s41598-020-78907-w

**Published:** 2020-12-11

**Authors:** Sidiki Malle, Milad Eskandari, Malcolm Morrison, François Belzile

**Affiliations:** 1 Département de Phytologie, Faculty of Agricultural and Food Sciences and Institute for Integrative and Systems Biology (IBIS), Laval University, Quebec City, QC Canada; 2 Department of Plant Agriculture, University of Guelph, Ridgetown, ON Canada; 3 Ottawa Research and Development Centre, Agriculture and Agri-Food Canada, Ottawa, ON Canada

**Keywords:** Computational biology and bioinformatics, Genetics, Molecular biology, Plant sciences

## Abstract

Soybean is an important source of protein, oil and carbohydrates, as well as other beneficial nutrients. A major function of proteins in nutrition is to supply adequate amounts of amino acids. Although they are essential for human nutrition, the sulfur-containing amino acids cysteine (Cys) and methionine (Met) are often limited and the genetic control of their content in soybean seeds is poorly characterized. This study aimed to characterize the phenotypic variation and identify quantitative trait loci (QTL) associated with Cys and Met content in a core set of 137 soybean lines, representative of the genetic diversity among Canadian short-season soybean, spanning maturity groups 000-II (MG000-II). Significant phenotypic differences were found among these lines for Cys, Met and Cys + Met content. Using both a mixed linear model and six multi-locus methods with a catalogue of 2.18 M SNPs, we report a total of nine QTLs and seventeen QTNs of which seven comprise promising candidate genes. This work allowed us to reproducibly detect multiple novel loci associated with sulfur-containing amino acid content. The markers and genes identified in this study may be useful for soybean genetic improvement aiming to increase Cys and Met content.

## Introduction

Soybean (*Glycine max* (L.) Merr) is a legume species native to East Asia and now widely planted for its edible bean, which plays an essential role in global food security^1^. Although soybeans are classified as an oilseed with 18–23% oil on a moisture-free basis, its increased production worldwide can be attributed also to its high protein content ranging 38–44%^2^. Soybean seed proteins are categorized as either albumins or globulins based on their solubility patterns^3^. The main storage protein is comprised of the globulin fraction, accounting for roughly 70% of the total seed protein. The 7S (β-conglycinin) and 11S (glycinin) polypeptides are the major components of the globulin fraction. This fraction provides 18 amino acids, including all 10 essential amino acids, but the sulfur-containing amino acids cysteine (Cys) and methionine (Met) are not present at adequate levels for optimal growth and development of monogastric animals^4^.

Cysteine and methionine contribute highly to human health and animal nutrition by preventing cancer, the development of cardiovascular diseases and promoting proper function of the immune system^5^. Unlike fat and starch, the body does not store excess amino acids for later use. Therefore, amino acids must be obtained from food daily^6,7^. Considering their role in maintaining human and animal health and their low level in soybean protein (< 3% of all amino acids in seed), developing soybean varieties with improved cysteine and/or methionine content is an important breeding objective to address soybean nutritional limitations^8^. Understanding the precise genetic architecture of these traits and identifying key genes controlling the accumulation of these protein fractions or amino acids would be an important step in this direction. So far, fifteen genes encoding β-conglycinins *(CG1–CG15)* and five genes (*Gy1–Gy5)* encoding glycinins have been reported^9,10^ (http://www.soybase.org/).

Seed content in sulfur amino acids has been shown to be quantitatively inherited and controlled by multiple genes^3,6^. Only a few studies have reported quantitative trait loci (QTL) associated with cysteine and methionine content in soybean seed (SoyBase, https://soybase.org/). Qiu et al.^11^ predicted 12 candidate genes based on the synteny between 113 genes from a gene mining of sulfur-containing amino acid metabolic enzymes in soybean. These authors also identified many QTLs related to the 7S (β-conglycinin), 11S (glycinin) fractions and Cys/Met content soybean on chromosomes 1, 3, 4, 6, 10, 13, 16, 17, 19 and 20. From linkage mapping, Panthee et al.^6^ reported a total of seven QTLs associated with Cys, Met and Cys + Met content across five chromosomes (Gm01, 07, 13, 17 and 18) in a population of 101 F_6_-derived recombinant inbred lines (RILs) (N87-984-16x TN93-99). Using the same population, Panthee et al.^12^ reported three QTLs associated with glycinin (Satt461, Satt156 and Satt292), on chromosomes (Gm17, 19 and 20) respectively, whereas two QTLs were detected for conglycinin (Satt461 and Satt249) distributed on chromosomes 17 and 16. From a bulked segregant analysis in the F_2_ mapping population derived from the cross of Harovinton and SQ97-0263_3-1a, Boehm et al.^13^ reported one QTL on chromosome 10 associated with the 7S α′ subunit and five QTLs associated with 11S A-type storage protein subunits respectively on chromosomes (Gm03, 10, 13 and 19). Fallen et al.^14^ detected two QTLs on chromosomes (Gm13 and 20) associated with both Cys and Met content in progeny of Essex x Williams 82 (282 F_5:9_ RILs). All these QTLs were reported as a result of QTL mapping in biparental populations and resulted in a poor overlap between the QTLs/QTNs reported in these studies. This is not unexpected since in biparental populations only two sources of allelic variation are evaluated. Moreover, the major limitation of this approach is that they offer poor resolution in detecting QTLs^15^ and the large confidence intervals surrounding the detected QTLs limits their utility for identifying candidate genes^16^.

Population-based mapping approaches such as genome-wide association studies (GWAS) can overcome many of these shortcomings of the biparental mapping approach^17^. In GWAS, we evaluate the association between each genotyped marker and a phenotype of interest that has been scored in a large number of unrelated individuals^18^. A GWAS can be achieved using a single-locus method where markers are tested individually in a one-dimensional genome scan like the Mixed Linear Model (MLM^19^) or using a multi-locus method where effects of all potentially associated markers are simultaneously estimated in a multi-locus genetic model such as the multi-locus random-SNP-effect Mixed Linear Model (mrMLM^20^). Furthermore, the use of next-generation sequencing in the context of GWAS makes it possible to genotype larger populations of plants with a higher density of markers than was previously possible and this contributes directly to increasing GWAS resolution.

With a single-locus approach, Vaughn et al.^17^ performed a GWAS on two different panels (IL-1996 and MS-1997), exploiting data from the Germplasm Resources Information Network (GRIN; https://www.ars-grin.gov/). The IL-1996 panel was composed of 900 cultivars belonging to maturity groups (MGs) III and IV, while the MS-1997 panel was composed of 978 cultivars from MGs V through IX. All the lines were genotyped using the SoySNP50K iSelect Array^17^ to produce a total of 32 K SNPs with a minor allele frequency (MAF) ≥ 0.05. This allowed these authors to identify a total of fourteen QTLs across 10 chromosomes (Gm01, 03, 02, 05, 06, 08, 10, 11, 16 and 20).

Recently, Lee et al.^21^ used both MLM and a Multi-Locus Mixed Linear Method (MLMLM) to conduct a GWAS for soybean seed components (including protein, oil, five fatty acids and 18 amino acids) on 313 plant introductions (PIs). These accessions belonged to MGs 0 (92%) and 00 (8%) and 91% originated from China. They were genotyped with the SoySNP50K Array and a total of 32 K SNPs with MAF ≥ 0.05 were used for association analyses. Only one QTL on chromosome 15 was associated with both cysteine and methionine. Using the same approach, Song et al.^19^ conducted a GWAS using phenotypic data collected from five environments for 621 accessions in maturity groups I–IV and 34,014 markers genotyped with the SoySNP50K array to identify one QTL on chromosome 3 and three (on Gm01, 15 and 18), respectively, associated with cysteine and methionine.

None of the previous work resulted in the identification of highly promising candidate genes that could be targeted by breeders for developing cultivars with increased content in sulfur-containing amino acids.

Thus, to obtain a comprehensive understanding of the genetic architecture of Cys and Met content in soybean seeds, we used a core set of 137 Canadian soybean lines grown on two sites and four replicates in total. The best linear unbiased predictor (BLUP) value of each line and a set of 2.18 M SNP markers [obtained from a combined dataset of genotyping-by-sequencing (GBS) and whole-genome sequencing (WGS)] were used to identify marker-trait associations. Both a single-locus method (MLM^19^) and six multi-locus GWAS methods (FASTmrEMMA^22^, FASTmrMLM^23^, ISIS EM-BLASSO^24^, mrMLM^20^; pKWmEB^25^ and pLARmEB^26^) were used. Here, we report a total of nine QTLs detected by the MLM method and seventeen major QTNs detected by at least two of the six multi-locus method located on 12 soybean chromosomes. In addition, seven strong candidate genes based on their high degree of association and functional annotation were identified.

## Results

### Phenotypic variation and correlations among traits

**Figure 1:**
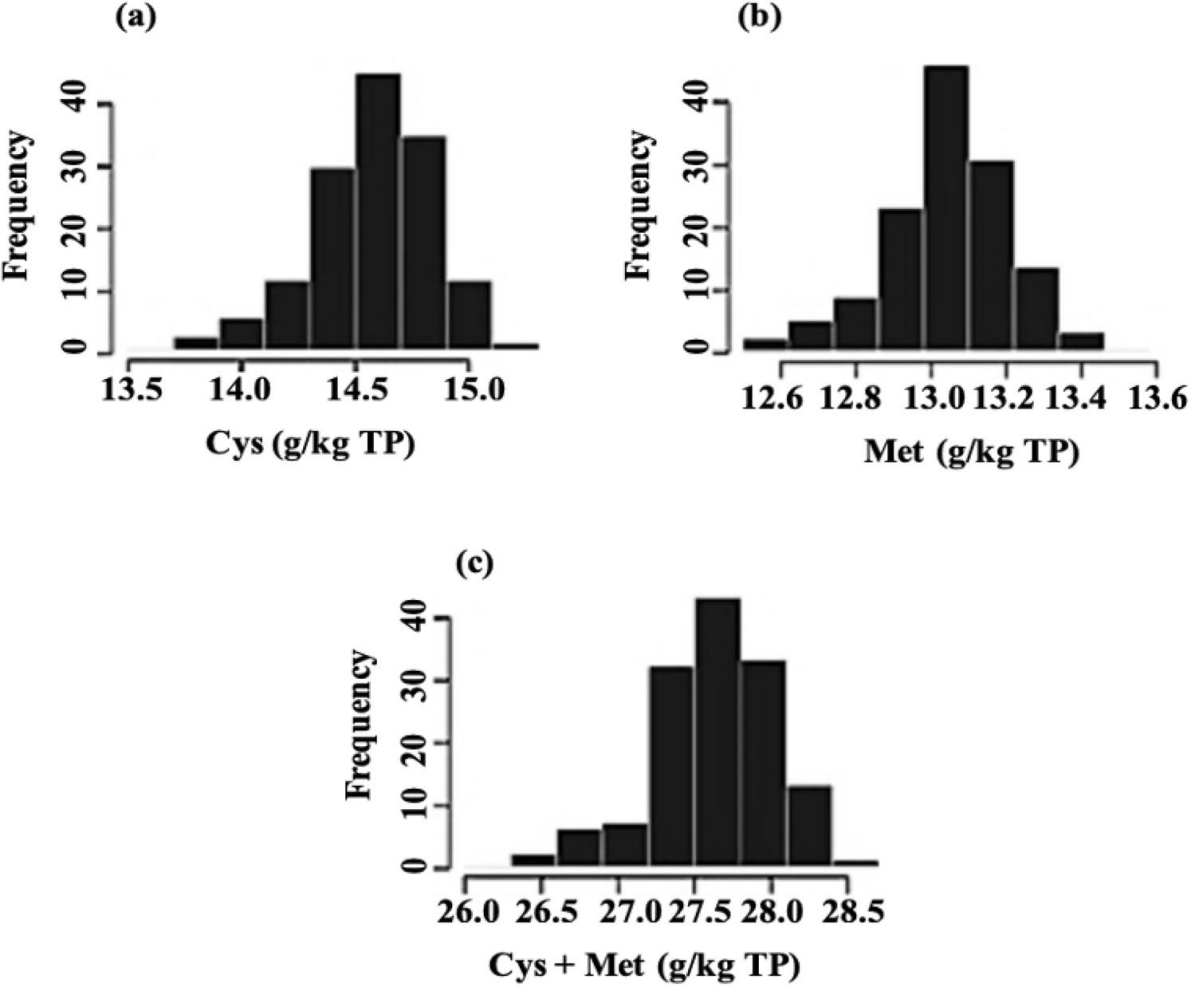
Distribution of Cys (**a**), Met (**b**) and (**c**) Cys + Met content (g/kg of total protein) in the seed of 137 Canadian soybean lines.

In view of performing a GWAS, we obtained phenotypic data for the sulfur amino acid content (g/kg TP) on a set of 137 soybean lines by growing single-row plots on two sites (two replicates/site) in 2013. The frequency distributions for Cys, Met and Cys + Met contents are presented in Fig. 1. All of the seed composition traits examined in this study exhibited a normal distribution as the *p*-value of a shapiro test for the three traits was < 0.05 and appeared to be quantitatively inherited. Descriptive statistics for Cys, Met and Cys + Met contents are presented in Table 1. It can be seen that the amino acid content varied from 13.5 to 15.0 g/kg on total protein basis (TP) for Cys and from 12.6 to 13.6 g/kg TP for Met. The total sulfur amino acid content Cys + Met varied from 26.0 to 28.5 g/kg TP. Across all 137 lines, the means were 14.6, 13.0 and 27.6 g/kg TP for Cys, Met and Cys + Met, respectively. The least significant difference (LSD) between two genotype means was 0.3 for Cys, 0.2 for Met and 0.5 g/kg TP for Cys + Met contents. An analysis of variance showed that the genotype main effect was a much greater source of variation *p* ≤ 0.001 than the environment and genotype by environment effect, but the latter was found to be a more important source of variation (*p* ≤ 0.01) for the sulfur amino acid content than the site. A high broad-sense heritability was observed and ranged from 49 to 62%.

**Table 1:** Descriptive statistics for cysteine, methionine and the sulfur amino acid content across two sites (two replicates per site).

Variable	Min	Max	Mean	LSD	Var (G)	Var (E)	Var (G x E)	H^2^ (%)
**g/kg of total protein**								
Cys	13.8	15.2	14.6	0.3	0.0002***	0.000008^NS^	0.00010**	49
Met	12.5	13.4	13.0	0.2	0.0001***	0.000014^NS^	0.00007**	62
Cys + Met	26.3	28.5	27.6	0.5	0.0009***	0.000047^NS^	0.00040**	57

LSD at the 0.05 probability level. Var(G) = genotypic variance, Var(E) = environmental variance, Var(GxE) = Genotype by environment variance and H^2^ = Broad sense heritability.

*NS* Not significant at α = 0.05.

**, ***Significant at the 0.01 and 0.001 probability levels, respectively.

To assess if these three traits were associated, we measured the correlation between them across the two environments. All three amino acid contents were positively and highly significantly correlated (*p* < 0.001). The Pearson correlation coefficients between these traits ranged from 0.81 to 0.97 (Table S1, upper diagonal) and the genetic correlation explaining the proportion of variance that two traits shared due to genetic causes ranged from 0.89 to 0.97 (Table S1, bold lower diagonal).

### Genotyping and SNP calling

Using the Fast-GBS pipeline for SNP calling, 93% of previous GBS single-end reads (~ 203 million 100-bp Illumina HiSeq2000) were successfully mapped onto the Wm82.a2.v1 reference genome. A total of 56 K high-quality SNPs were obtained and so called «GBS-derived SNP». To obtain an extensive SNP coverage of the genome, we used a two-step approach to genotype the 137 lines of this association panel (Fig. S1). In a first step, we re-analyzed previously obtained reads derived from GBS analysis to take advantage of an improved SNP-calling pipeline and reference genome. Overall, 93% of ~ 203 million 100-bp Illumina HiSeq2000 single-end reads successfully mapped onto the reference genome and 56 K high-quality GBS-derived SNPs were identified using the Fast-GBS pipeline (see “Materials and methods” section). In a second step, to further densify marker coverage, we performed the imputation of close to 4.3 M SNPs by using a set of 102 resequenced lines (of which 56 were common to both sets of lines) as a reference panel. This fully imputed set of 4.3 million SNPs was filtered to remove InDels and to retain markers with a MAF ≥ 0.05 and heterozygosity ≤ 0.1. This yielded a catalogue of 2.18 M SNPs distributed across the twenty chromosomes (Fig. S2), an average marker density of 1 SNP every 435 bases across the entire genome.

### Population structure

**Figure 2:**
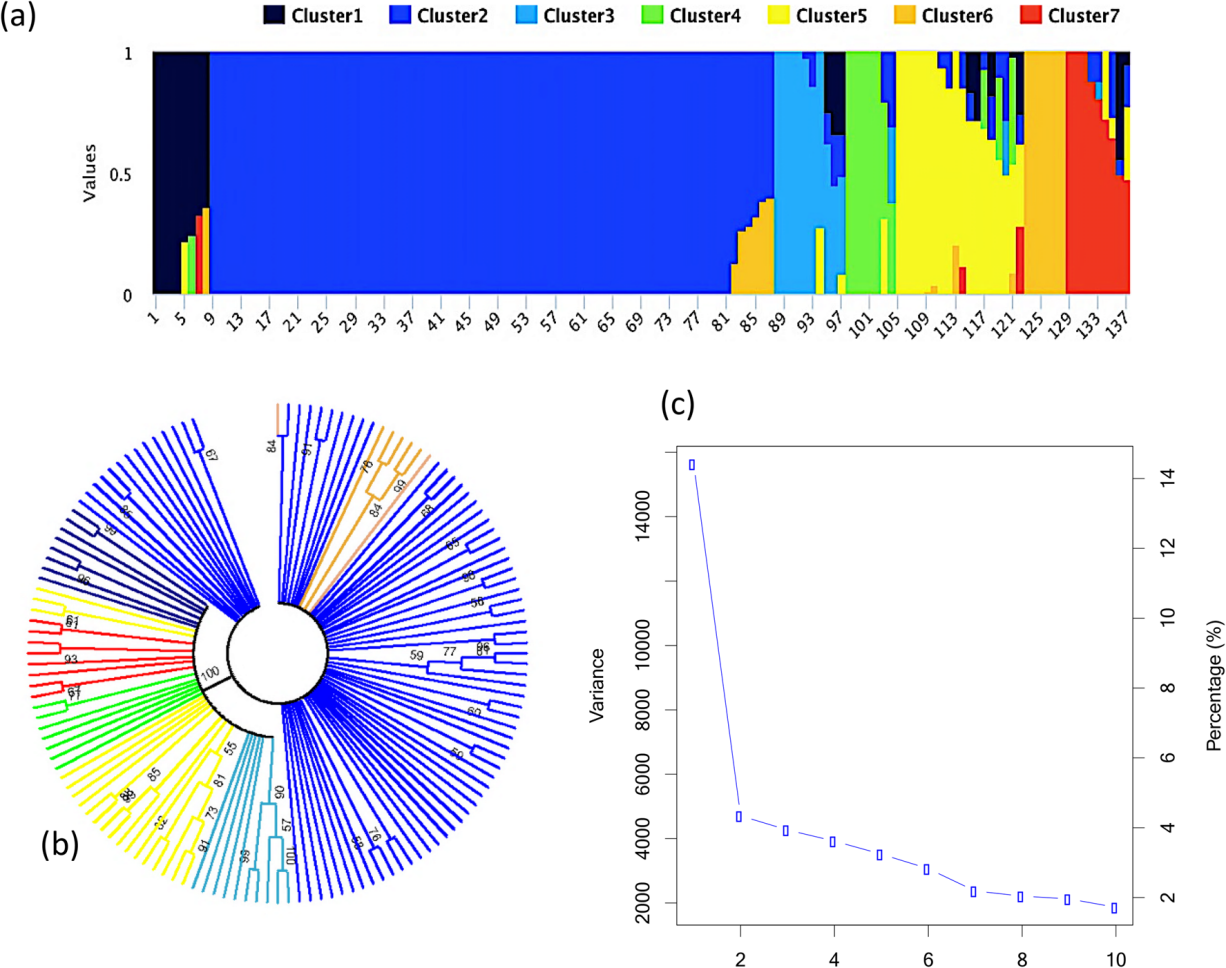
(**a**) Classification into seven populations using fastSTRUCTURE where each individual (from 1 to 137) is represented by a single vertical line and each color represents one cluster. (**b**) Bootstrap consensus phylogenetic tree (2000 replicates) constructed using MEGA 7; each color represents a subgroup and seven subgroups were found in total and (**c**) PCA eigenvalues computed using GAPIT.

Based on a posterior Bayesian clustering analysis performed using a subset of pruned markers (r^2^ ≤ 0.5), the optimum number of subpopulation (*k*) was 7 (Fig. 2a). A phylogenetic tree in (Fig. 2b) constructed with the same subset of markers showed seven main branches with bootstrap values ≥ 50%. Similarly, for PCA (Fig. 2c), the total variance explained by each principal component (PC) decreased from PC1 to PC7 and, after PC7, the variance explained by each further PC remained low and stable. Together, these results suggested that *k* = 7 provided a good assessment of population structure (illustrated in Fig. 2). These subpopulations tended to contain lines belonging to the same maturity group and/or originating from the same breeding programs and the corresponding Q matrix was used for GWAS.

### Genome-wide association scan for sulfur amino acid content

To identify genomic regions associated with the seed content in sulfur amino acids, a genome-wide scan was performed using a MLM and six multi-locus models implemented in the mrMLM package v4.0. A pruned set (r^2^ ≥ 0.9) of SNP markers and a single phenotypic value (BLUP) for each trait were used.

**Figure 3:**
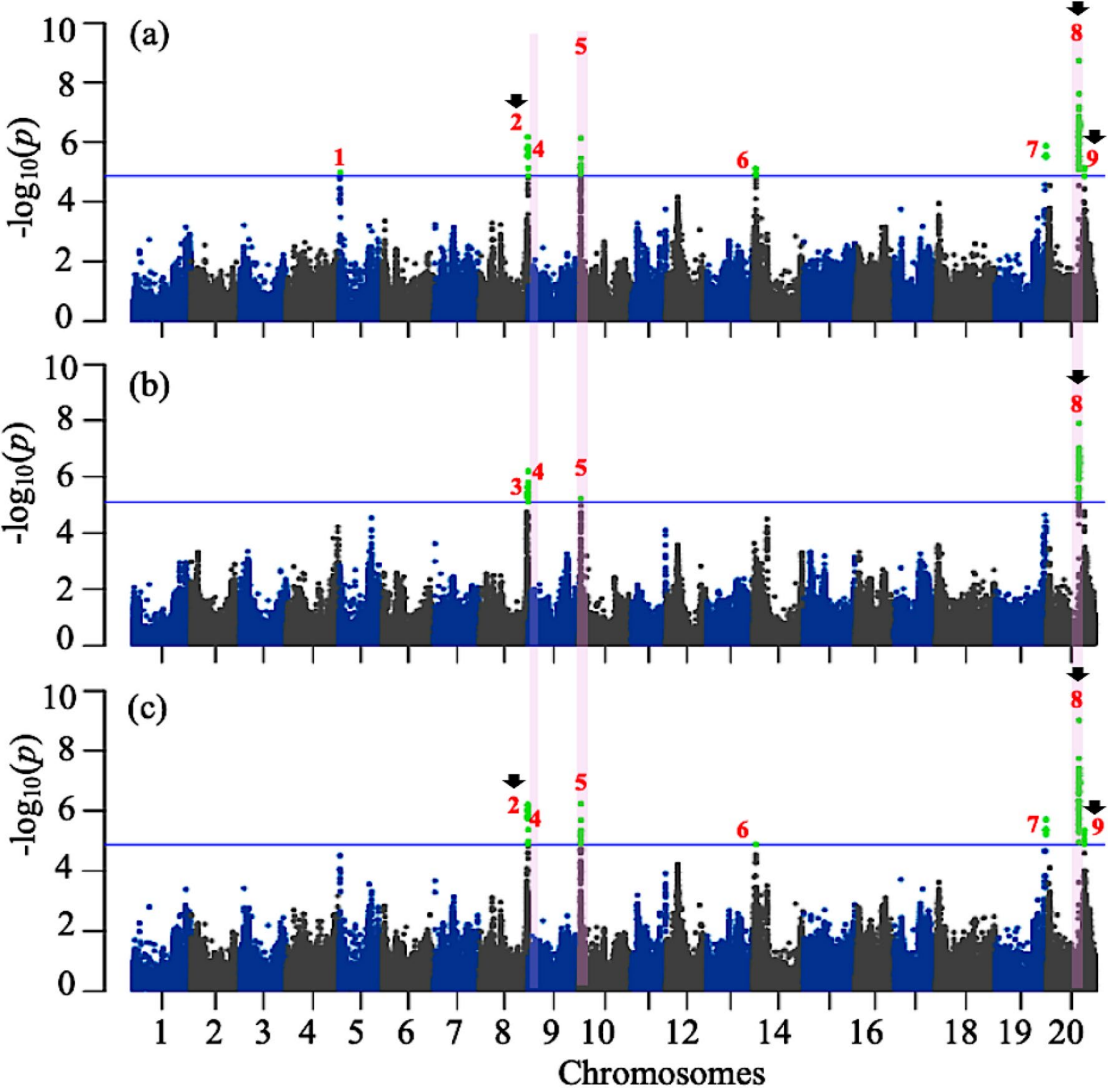
Manhattan plots for (**a**) Cys, (**b**) Met, and (**c**) Cys + Met content in a core set of 137 Canadian soybean accessions using MLM.

For the MLM analyses, as can be seen in the Q–Q plots shown in Fig. S3, this method successfully limited the confounding effects of population structure and kinship as the observed *p*-values only diverged from the diagonal (expected *p*-values) at the most extreme values (beyond 3E−03 for all three traits). The results of these MLM association analyses are presented as genome-wide Manhattan plots in Fig. 3. With the chosen threshold for false discovery rate (blue horizontal line, FDR ≤ 0.05), only 0.03% of the SNPs (71 of the 243 K) were significantly associated with Cys content, with the uncorrected *p*-values ranging from 1.37E−05 to 1.82E−09. There were 41 markers (0.02%) significantly associated with Met content (*p-*value from 8.02E−06 to 1.25E−08) and 71 SNPs (0.03%) with Cys + Met content (*p-*value from 1.32E−05 to 9.56E−10) as uncorrected *p-*values.

To identify distinct QTLs, we characterized haplotype blocks formed by these associated SNPs according to Gabriel et al.^27^. For SNPs associated with more than one trait, the most significant SNP (lowest FDR, across all significantly associated traits) was retained to mark a given haplotype block. In total, nine haplotype blocks (numbered 1–9 in Fig. 3) were defined of which eight were associated with Cys, four with Met and seven with Cys + Met. In three cases, the same QTL/haplotype block was detected for all three traits (QTLs #4, 5 and 8 on Gm08, 10 and 20) and in four cases, the same QTL was shared between two of the three traits, Cys and Cys + Met (QTLs #2, 6 ,7 and 9 on Gm08, 14, 19 and 20). Only two QTLs (QTLs #1 and 3 on Gm05 and 08) were unique to a single trait (Cys and Met, respectively). The features of these nine QTLs are summarized in Table 2. The portion of phenotypic variance explained (R^2^) varied between 15 and 27%. The magnitude of allelic effects were calculated according as per Kang et al.^28^ and varied between 0.12 and 0.42 g/kg TP.

**Table 2:** The most highly associated SNP markers within each QTL from MLM analyses and their effects on phenotypic variation for sulfur amino acid content in a Canadian soybean collection.

Trait	Chr	Peaks SNP (bp)	QTL #	*p*-values	FDR	R^2^	Allelic effect
Cys	5	837,667	1	1.04e−05	0.039	0.16	0.24
Cys/Cys + Met	8	47,147,391^a^	2	6.73e−07	0.007	0.21	0.27
Met	8	47,343,134	3	2.18e−06	0.022	0.17	0.16
Cys/Met/Cys + Met	8	47,797,482	4	1.65e−06	0.012	0.19	0.26
Cys/Met/Cys + Met	10	1,815,572	5	5.93e−06	0.036	0.16	0.15
Cys/Cys + Met	14	1,755,083	6	1.32e−05	0.045	0.15	0.42
Cys/Cys + Met	19	49,914,926	7	1.95e−06	0.011	0.19	0.42
Cys/Met/Cys + Met	20	31,861,877^a^	8	1.25e−08	0.003	0.27	0.22
Cys/Cys + Met	20	37,038,638^a^	9	7.11e−06	0.030	0.16	0.12

^a^QTLs codetected by MLM and the multi-locus methods.

**Figure 4:**
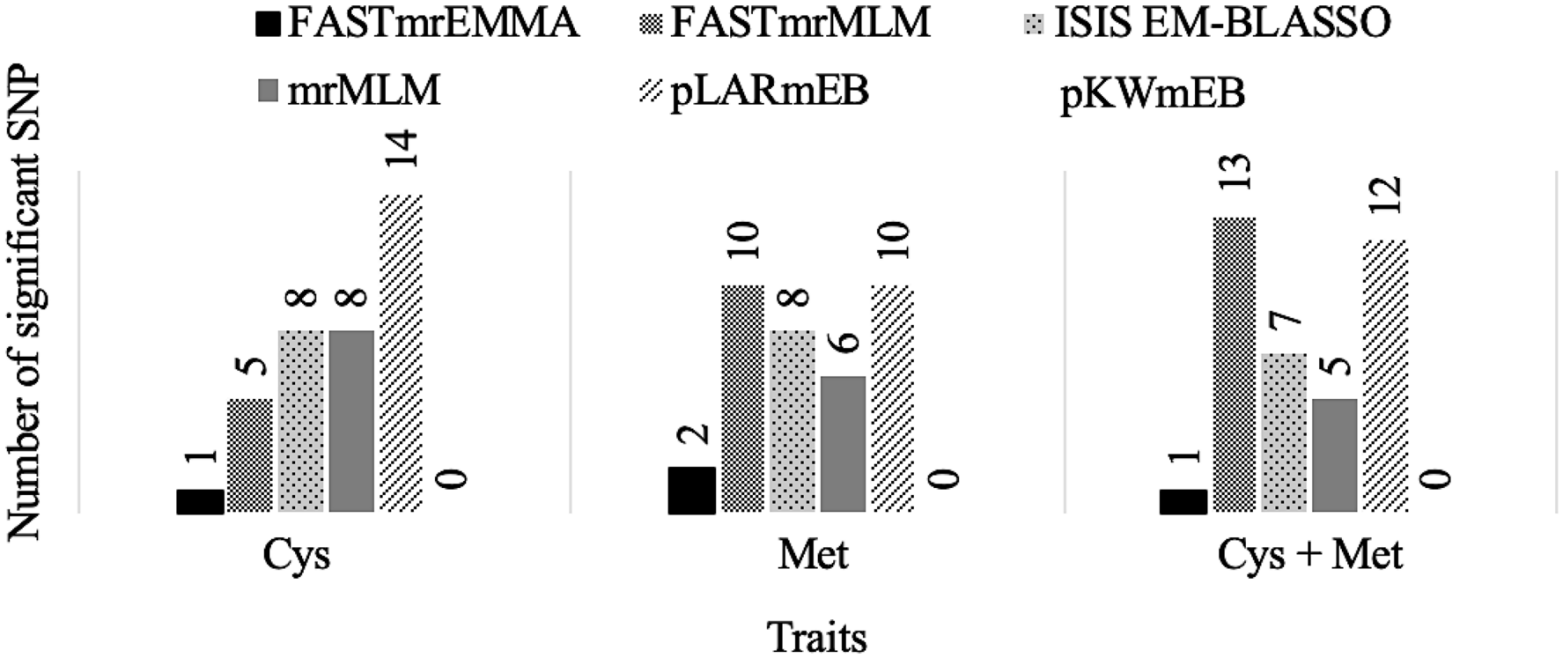
Total number of significant SNPs detected using six multi-locus methods.

From the multi-locus analyses, ~ 0.01% of the SNPs (36, 36 and 38 of the 243 K) were significantly associated with Cys, Met and Cys + Met content, respectively. The LOD score of these associated SNPs ranged from 3.26 to 17.65 for Cys, 3.05 to 18.98 for Met and 3.04 to 13.15 for Cys + Met content. Across the six multi-locus methods (Fig. 4), pKWmEB detected the fewest associated SNPs (0), while the highest number was detected by pLARmEB (14 SNPs associated with Cys content).

Here, we report QTNs that met the following criteria: (i) the associated SNP was detected by at least two multi-locus methods and (ii) explained at least 5% of the phenotypic variance (R^2^). In total, seventeen QTNs/haplotype blocks were identified in this fashion including three QTNs codetected by both MLM and multi-locus methods (Table S2). In most cases, using multi-locus methods, the exact same SNP was the most highly associated SNP with each trait, although occasionally, a different SNP residing in the same haplotype block was most highly associated (e.g. Gm08: 43,871,472 for ISIS EM-BLASSO and Gm08: 43,877,254 for mrMLM). In the latter case, to be conservative, the SNP with the lowest LOD score was reported as the QTN. Of these seventeen QTNs, four (Cys), three (Met) and five (Cys + Met) were unique to a single trait. One QTN was shared by each of two trait pairs (Cys/Met and Met/Cys + Met content), while three QTNs were detected for all three traits. The R^2^ of these multi-locus QTNs varied from 5.20 to 25.39% and the QTN effect varied between 0.03 and 0.31 g/kg TP.

Each dot indicates the degree of association between a single marker and a trait (y-axis) while the x-axis shows the physical position of each marker. A blue horizontal bar indicates the significance threshold (FDR ≤ 0.05) and significantly associated markers are coloured in green. The red numbers (from 1 to 9) identify the different QTLs based on the haplotype blocks formed by the significantly associated markers. The pink vertical stripes highlight QTLs that were common to all three traits and the black arrows indicates the overlapped QTL between MLM and multi-locus methods.

### MLM QTL validation

**Figure 5:**
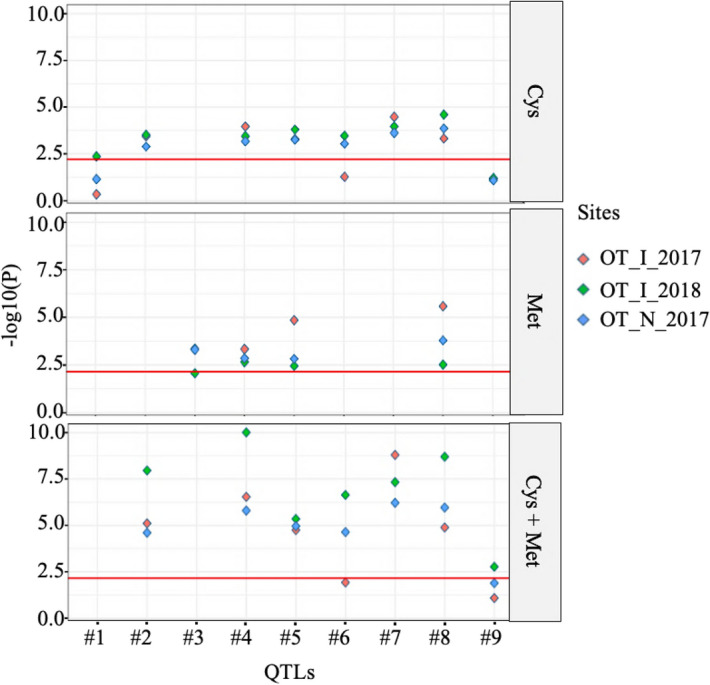
Degree of significance (*p*-value) of the contrast between phenotypic means for lines carrying different alleles at candidate QTLs for sulfur-containing amino acid content in whole soybean seeds. Cys, Met, and Cys + Met content was measured in a core set of 137 Canadian soybean accessions in three environments (indicated by coloured symbols). A t-test was performed to compare the mean trait value for lines sharing one or the other allele at the nine QTLs discovered in the MLM method. Each coloured symbol represents the *p*-value for this comparison of means. The red horizontal bar indicates the adjusted Bonferroni significance threshold at alpha ≤  − log10(0.05/8 QTLs) for Cys, alpha ≤  − log10(0.05/4 QTLs) for Met and alpha ≤  − log10(0.05/7 QTLs) for Cys + Met content.

To validate the QTLs discovered by the MLM method, data from three additional trials were obtained. Given the difference in original purpose and experimental design of this second set of trials, the resulting data were used to assess if the phenotypic mean differed between lines carrying one or the other allele at each QTL (Table S3). For Cys, seven out of the eight QTLs resulted in a significant difference (*p* < 0.006) in the mean Cys content between lines carrying contrasting alleles in at least one of the three validation trials. Six out of eight QTLs were validated in at least two trials and five QTLs were validated in all three trials. For Met, of the four QTLs discovered initially, all four were validated (*p* < 0.01) in all trials. For Cys + Met, all seven QTNs were validated (*p* < 0.007) in at least one trial, six were confirmed in at least two trials and five were validated in all three trials (Fig. 5). Together, these data strongly suggest that the QTLs identified through GWAS are robust and highly reproducible across multiple environments.

### Multi-locus QTN validation

To validate the QTNs discovered by the multi-locus methods, the same approach and phenotypic data used for validation of MLM-derived QTLs was used. With the exception of QTN_#7 in the OT_I_2018 environment, unique QTNs (i.e. not including the three coinciding with QTLs) did not produce a significant phenotypic contrast between lines carrying alternate alleles at these loci (Table S4). Thus, we find that the QTNs discovered using a multi-locus model could seldom be validated using an approach relying on the observation of phenotypic contrasts resulting from considering these loci individually.

### Prediction of candidate genes within the QTL/haplotype blocks regions

**Figure 6:**
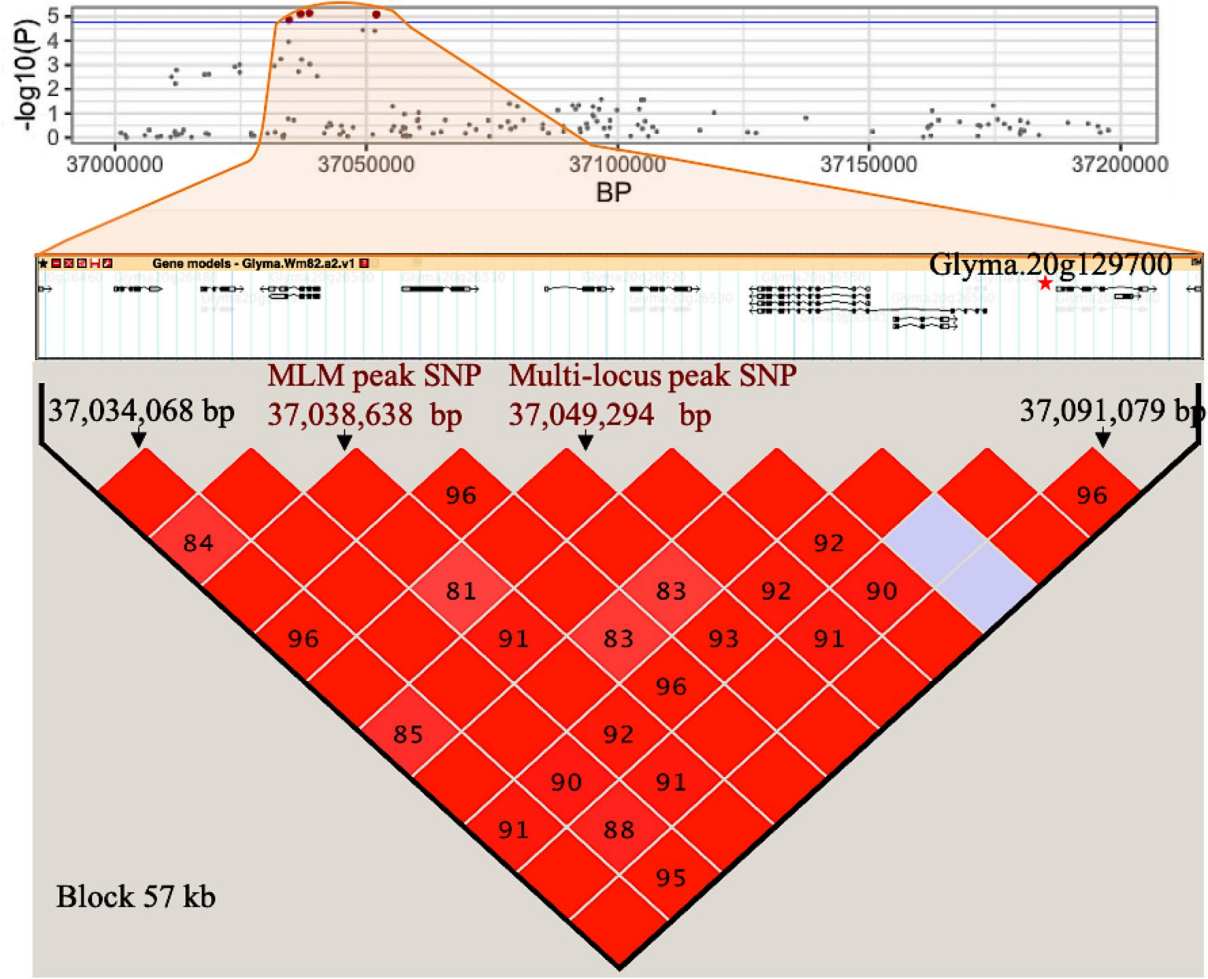
Identification of candidate genes within the haplotype block codetected by both MLM and the multi-locus methods (QTL #9 and QTN #17) on chromosome 20.

To establish a list of candidate genes, all genes residing within one of the haplotype blocks identified by MLM or the multi-locus methods (ranging in size between 5 and 376 kb) were extracted from SoyBase and their GO annotations examined. The number of genes per haplotype block varied between 1 and 32, and the full list of these genes and their annotations are provided in Table S5. An example of this approach is illustrated in Fig. 6. Among these haplotype blocks, the haplotype block codetected by MLM and by four of the six multi-locus methods (FASTmrMLM, ISIS EM-BLASSO, mrMLM and pLARmEB) corresponding to QTL #9 (MLM) and QTN #17 (multi-locus) spanned 64 kb and included 12 genes. Among these genes, Glyma.20g129700 was located 45 kb upstream of the MLM peak SNP (Gm20: 37,038,638) and 35 kb upstream of the multi-locus peak SNP (Gm20: 37,049,294). This gene was annotated as being involved in methionine biosynthesis (GO: 0009086). In addition, within haplotypes identified from MLM analysis, only one other candidate gene had an annotation evoking an association with sulfur amino acid content. Glyma.08g367600 is located 10.8 kb downstream of the peak SNP in QTL #4 (Gm08: 47,797,482), encodes a protein that is predicted to have an oxido-reductase activity and its annotation (GO: 0000096) suggests a role in sulfur amino acid metabolism.

Five other candidate genes were identified within four haplotype blocks identified only by the multi-locus methods and correspond to QTNs #1, #10, #11 and #14 (Table 3). Among them, Glyma.13g108800, Glyma.14g003200 and Glyma.14g003400 were all annotated as being involved in cysteine biosynthetis. Glyma.13g108800 was found among 11 other genes in a 71-kb haplotype block and located 17 kb uptream of the peak SNP corresponding to QTN #10 (Gm13: 22,275,813). The last two candidate genes were found within a haplotype block of 149 kb corresponding to QTN #11 and included 24 other genes. These were located 61 kb and 50 kb downstream of the peak SNP (Gm14: 367,224).

**Table 3:** Candidate genes underlying the reported QTLs/QTNs based on a relevant GO annotation.

Traits	Gm	Nbr. genes in HB	QTL	QTN	Candidate genes	Relevant annotation
Met	4	21	–	#1	Glyma.04g237300	Spermidine synthase activity
Cys/Met/Cys + Met	8	7	#4	–	Glyma.08g367600	Oxido-reductase activity
Cys	13	12	–	#10	Glyma.13g108800	Cysteine biosynthetic process
Cys/Met/Cys + Met	14	26	–	#11	Glyma.14g003200	Cysteine biosynthetic process
					Glyma.14g003400	Cysteine biosynthetic process
Met	16	7	–	#14	Glyma.16g032200	Ethylene biosynthetic process
Cys/Met/Cys + Met	20	12	#9	#17	Glyma.20g129700	Methionine biosynthetic process

Two other candidate genes, Glyma.04g237300 and Glyma.16g032200, had an annotation indirectly associated with the methionine biosynthesiss. They were located 43 kb and 23 kb uptream of the peak SNPs (QTN #1, Gm04: 50,542,407) and (QTN #14, Gm14: 3,028,024), respectively. Glyma.04g237300 was annotated to have a spermidine synthase activity and found among 21 other genes in a 243-kb haplotype block while Glyma.16g032200 was located in a 56-kb haplotype block containing 6 genes and is annotated as being involved in ethylene biosynthetiss.

**Figure 7:**
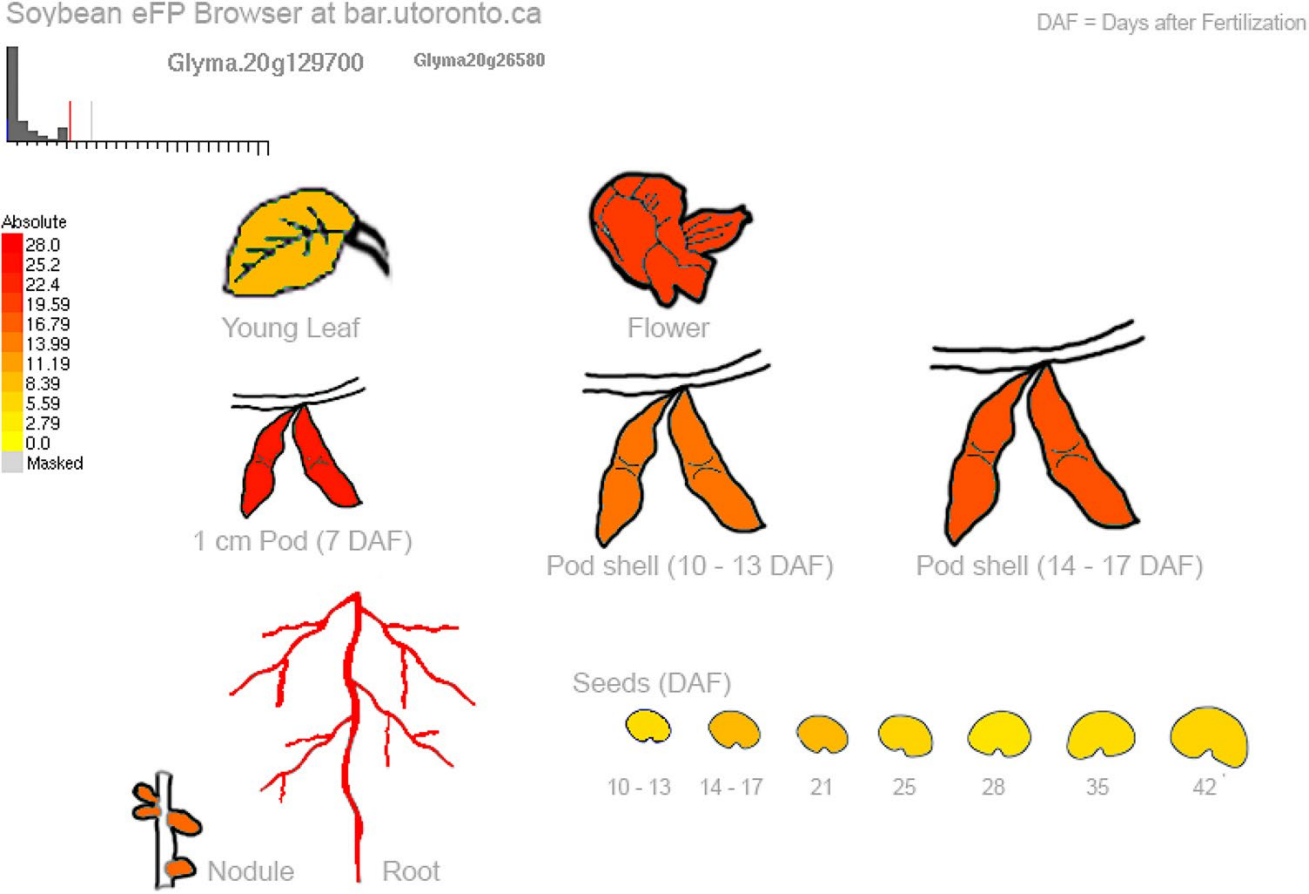
Expression profile of Glyma.20g129700 based on previous transcriptomic analysis in soybeans. Expression strength coded by color: yellow = low, red = high. As shown, Glyma.20g129700 is most highly expressed in flowers, pods, roots, pod shells and in the seed. Data derived from RNA-seq of *Glycine max*, published by Severin et al.^21^ and download from eFP browser^29^ (www.bar.utoronto.ca).

We then investigated the expression profile of these seven candidate genes. Based on previous transcriptomic analyses, these genes (except Glyma.08g367600) were known to be mainly expressed in young leaves, flowers and roots but also expressed in pods 7 days after flowering (DAF), pod shells (10–13 DAF) and in seeds (14–17 and 35 DAF) as illustrated in Figs. 7 and S4. There were no transcriptomic data available yet for the expression profile of Glyma.08g367600, likely due to the fact that it is present only in the current Wm82.a2 assembly/annotation.

Top panel constructed from MLM results shows the Manhattan plot for the marker-trait associations showing the region of interest in light orange on Gm20. The blue horizontal line represents the FDR threshold (FDR ≤ 0.05). The central panel shows the position and orientation of all ten gene models present in the haplotype block (bottom panel). The most likely candidate gene (Glyma.20g129700, involved in methionine biosynthetis) is highlighted with a red asterisk and is 45 kb and 35 kb upstream of the MLM peak SNP (Gm20: 37,038,638) and the multi-locus peak SNP (Gm20: 37,049,294), respectively. The extent of LD is indicated as D′ × 100. The purple squares indicate no LD (D′ = 0) while empty red squares indicate complete LD (D′ = 1).

### Structural and nucleotide variation within candidate genes and their predicted functional impact

Given the availability of WGS data for 56 lines of our association panel, we looked for structural or nucleotide variation within the eight candidate genes identified previously. No structural variants > 51 bp were identified in any of the candidate genes within these 56 lines. We also examined if SNP variants within coding regions could impact gene function. In total, 49 nucleotide variants were identified within coding regions of seven candidate genes. All of these variants were predicted as having a “low” to “modifier” effect. As such, we do not consider these variants to be likely causal.

## Discussion

### Phenotypic variation and correlations among traits

Normal distributions were observed for all the traits (Shapiro test p-value < 0.05) and suggested that these traits are controlled by polygenes. The phenotypic variation ranged from 13.5 to 15.0 g/kg for Cys and from 12.6 to 13.6 g/kg for Met content. The total sulfur amino acid content Cys + Met varied from 26.0 to 28.5 g/kg on the basis of total protein. The observed phenotypic values reported in this study were slightly lower but similar in range to those reported in previous studies when similarly expressed in terms of g of amino acid per kg of total protein. Warrington et al.^3^ reported 14.8 to 16 g/kg and 13.9 to 14.7 g/kg for Cys and Met content. Song et al.^19^ reported 12.1 to 14.5 g/kg and 11.5 to 13.7 g kg respectively for Cys and Met contents. However, in terms of range, the results reported here correspond most closely to those reported by^3^ (1.4 and 0.9 g/kg, respectively, for cysteine and methionine content). Moreover, similarly to previous studies, high and positive correlations (0.81 to 0.97) were seen between cysteine, methionine and the total sulfur amino acid contents. It has been suggested that the high phenotypic and/or genotypic correlations between cysteine and methionine contents are due to a shared biosynthetic pathway or a similar molecular basis of inheritance^6,30^.

### Genome-wide association analysis

For marker-trait association analysis, a total of 243 K SNP markers and a single phenotypic value for each line (BLUP) was used in two GWAS approaches (one single-locus (MLM) and six multi-locus (FASTmrEMMA, FASTmrMLM, ISIS EM-BLASSO, mrMLM; pLARmEB, and pKWmEB) methods). In all cases, we incorporated population structure and family relatedness to control for confounding effects. We reported a total of nine QTLs/haplotype blocks associated with the sulfur amino acid content detected by an MLM approach and seventeen major QTNs detected by at least two of the six multi-locus methods. Interestingly, three chromosomal regions were detected using both approaches. Among MLM-derived QTLs, one third of these SNPs were significantly associated with all three traits.

Importantly, approximately two thirds of these QTLs proved extremely robust as they could be successfully confirmed in three additional trials. This suggests that these individual markers would provide a good tool for breeders interested in using marker-assisted selection (MAS) to improve Cys and/or Met content within this germplasm. In contrast, however, the QTNs uncovered in the multi-locus analysis could not be validated in the same fashion. One must keep in mind though that these QTNs were discovered in a model based on the joint consideration of these loci whereas our validation experiment was based on the consideration of loci individually. This may have contributed to our inability to confirm the impact of the QTNs on these traits. Nonetheless, in the context of MAS, it is likely that these QTN would not prove useful; these might be better captured in a genomic-selection scheme relying on a wider set of loci and allowing for multi-locus contributions to the phenotype.

In previous mapping work, the total number of QTLs associated with cysteine and methionine content varied from 1^31^ to 14^17^ in GWAS studies while 2^14^ and 8 QTLs^32^ were reported following family-based mapping. As we observed here, a subset of QTLs were common to both cysteine and methionine content^6,7,31^ and, in one case, all the QTLs (eight) were associated with both cysteine and methionine content^32^. This is unsurprising given the high degree of correlation between these traits and the fact that both amino acids share the same biosynthetic pathway with cysteine being a precursor of methionine production^33^.

To compare the genomic regions reported by previous studies with the QTLs reported in this study, we used SoyBase to define the physical position of previously reported QTLs. Two of the three QTL/QTN codetected by both approaches in this study fell within genomic intervals identified in previous biparental mapping work. Indeed, Wang et al.^32^ reported one QTL Cys 3–7 located from 3.0 to 35.9 Mb on chromosome 20 associated with seed cysteine content. Moreover, this genomic region fell within QTL qMet_Gm20 and qCys + Met_Gm20 (24.5 to 34.7 Mb) reported by Warrington et al.^3^ associated with Met and Cys + Met content. Interestingly, this genomic region overlapped with QTL #8 from MLM analysis and QTN #16 from multi-locus analysis located on chromosome 20 at 31.8 Mb. Similarly, Ramamurthy et al.^34^ reported one QTL on chromosome 20 flanked by *BARC-038869–07364* and *BARC-039753–07565* (36.5 to 39.8 Mb) simultaneously associated with both seed Cys and Cys + Met content, an interval within resides a second codetected QTL, (QTL/QTN #9/#17 for MLM/ multi-locus) on chromosome 20 (Gm20, 37.0 Mb).

None of the seven other QTLs that we reported from MLM analysis in this study coincided with previously reported QTLs intervals identified in family-based mapping. Similarly, none of the QTLs reported by previous GWAS studies^17,31,35^ were located within 500 kb of those reported in this study. Except for the work of Zhang et al.^31^ in which only one QTL was reported, all other GWAS studies have been conducted within later maturity groups (MG I—IX) and the limited overlap in QTLs could be attributed to differences in the loci controlling these traits within the different maturity groups.

Considering that sulfur amino acids are components of protein fractions, notably the glycinin (11S) and conglycinin (7S) storage proteins, one might expect that genomic regions reported as significantly associated with sulfur amino acid content also be associated with the abundance of 11S and 7S, which together constitute nearly 70% of total storage proteins in the soybean seed^12^. Interestingly, Ma et al.^36^ reported two QTLs significantly associated with glycinin and beta-conglycinin content on chromosome 10 (174 kb and 2.7 Mb) and 20 (34.6 to 37.8 Mb) a genomic region spanning QTL #5 and #9 reported in this study (Gm10 at 1.8 Mb) and (Gm20 at 37.0 Mb) significantly associated with all three traits.

For the multi-locus analyses, we reported a total of seventeen QTNs across twelve chromosomes including three shared regions with the MLM method. Across the traits, three QTNs were detected for all three traits. Two QTNs were shared by two trait pairs (one each for Cys/Met and Met/Cys + Met content). Four (Cys), three (Met) and five (Cys + Met) were unique to a single trait. Similar to the QTLs discovered via MLM, very little overlap was observed between previously reported QTLs and the multi-locus QTNs identified in this study. Among the seventeen QTNs, QTN #10 located on chromosome 13 (Gm13, 22.2 Mb) coincided with one QTL reported by Qiu et al.^11^ to be significantly associated with cysteine content (flanked by BARC-042289-08234 and BARC-045205-08910*)*.

Four of the QTNs reported in this study (QTN #1 and #2 at 48.4 and 50.5 Mb on Gm04; QTN #14 at 3.0 Mb on Gm16 and QTN #17 at 37.0 Mb on Gm20) were significantly associated with all three traits and these coincide with three broad genomic regions reported by Ma et al.^36^ that were significantly associated with seed glycinin, beta-conglycinin and seed glycinin to beta-conglycinin ratio content across three chromosomes (Gm04: 7.6 to 51.2 Mb, Gm16: 569 kb to 29.5 Mb and Gm20: 34.6 to 37.8 Mb).

### Candidate genes and their functions for sulfur amino acid accumulation

The peak SNPs associated with sulfur amino acid content reside in the nine and seventeen haplotype blocks identified through MLM and multi-locus analysis within which one would expect to find the causal genes that control Cys and Met content in this collection of soybean lines. Of the genes located within these haplotype blocks, seven stood out as promising candidate genes based on their GO annotations.

Of these seven candidate genes, only Glyma.20g129700 was identified using both MLM and multi-locus analysis and encodes a 5-methylthioribose kinase protein. This protein was demonstrated to be a primary enzyme involved in the recycling of the methylthio group of 5-methylthioribose back into methionine^37,38^.

A second promising candidate gene identified using MLM, Glyma.08g367600, encodes a protein that is predicted to have an oxido-reductase activity acting on NAD(P)H. Although the annotation for this gene indicates a role in sulfur amino acid metabolism, we have not been able to pinpoint the precise role of the protein encoded by this gene in sulfur amino acid metabolism. Moreover, no information from previous studies or transcriptomic data was available for this gene. Its role in the metabolism of sulfur amino acids needs to be confirmed.

Among the five other candidate genes identified from multi-locus analysis, Glyma.04g237300 was annotated as a spermidine synthase and said to be involved in methionine synthesis in plants. From the work of Qiu et al.^11^, the spermidine synthase encoding gene was found in association with eleven other genes to contribute 6–38.5% of the genetic variation of sulfur-containing amino acids.

Glyma.13g108800 encode a stress-responsive alpha–beta barrel protein and is thought to be involved in both cysteine and methionine synthesis under stress conditions. Cysteine-containing molecules like glutathione and thioredoxin play a major role in maintaining an intracellular reducing environment and protecting the cell from oxidative damage^39^.

Glyma.16g032200 encodes a protein that is involved in ethylene biosynthetis and in the l-methionine salvage cycle II (in plants). The ortholog of this gene corresponds to the *A. thaliana AT3G61510.1* locus, encoding an ACC synthase 1. In plants, the methionine is a precursor of ethylene synthesis. Therefore, this biosynthetic pathway is directly connected to the methionine cycle^40,41^.

Although Glyma.14g003200 and Glyma.14g003400 were both annotated as being involved in cysteine biosynthesis, again, no direct link was found between these genes and cystheine biosynthesis through previous studies.

As none of the SNPs located inside the coding region of our candidate genes were predicted to cause a functional change, it is likely that the alleles of this gene differ in expression, thus resulting in the observed differences in sulfur-containing amino acids. Taken together, these data strongly suggest that the phenotypic variation observed in this collection of Canadian lines is due to variants located outside the coding region that likely result in differences in gene expression.

## Materials and methods

### Plants material and field trials

A set of 137 lines thought to constitute a core set representative of the extent of genetic diversity among soybean varieties belonging to maturity group 000-II ( MG000-II) in Canada was selected from a larger group of 304 accessions based on the analysis of population structure as previously described in^42^. Plant research material was originally provided by four breeding programs, respectively CÉROM at St-Mathieu-de-Beloeil (QC), Agriculture and Agri-Food Canada at Ottawa (ON), University of Guelph (ON) and Semences Prograin Inc at St-Césaire (QC). These lines were grown in field trials conducted on two research farms, namely Woodstock (ON, 43.1447° N, 80.7809° W) and St-Mathieu-de-Beloeil (QC, 45° 35′ 14.1′′ N 73° 14′ 59.6′′ W) in Canada in 2013. The experimental design was a generalized lattice in which all lines were planted in either 4-m rows with 50-cm spacing or 6-m rows with 30-inch spacing in Woodstock and St-Mathieu-Beloeil, respectively, with two replicates at each site.

### Phenotyping and statistical analysis

For sulfur amino acid content for both mapping and validation population, 25-g samples (from each replicate) of whole seeds were analyzed using near-infrared spectroscopy (Perten NIR DA 7250, Perten Instruments). The NIR used was calibrated according to manufacturer’s calibration module based on set of 2646 samples. After sample scanning, the concentration of sulfur amino acids was expressed on the basis of the dry matter percentage. Each value was then normalized per kg of total protein (g/kg TP). Three traits were considered in the ensuing analyses: cysteine content (Cys), methionine content (Met) and the sum of both (Cys + Met). Statistical analyses on amino acid content data were performed using META-R^43^. Descriptive statistical analysis and a mixed linear model were used to determine genotypic variance, environment and genotype × environment effects as well as a Shapiro–Wilk test for normal distribution of the phenotype. The two sites were considered as two environments and all effects were assumed random, the broad-sense heritability for a given trait across the two environments was calculated as follows:

$${H}^{2}=\frac{{\sigma }_{\text{g}}^{2}}{{\sigma }_{\text{g}}^{2}+{\sigma }_{\text{g}e}^{2}/nEnv+{\sigma }_{e}^{2}/(nEnv \times nrep) },$$

where ${\sigma }_{\text{g}}^{2}\, \text{and}\, {\sigma }_{\text{g}e}^{2}$ are the genotype and genotype × environment interaction variance component, $nEnv$ is the number of environments,${\sigma }_{e}^{2}$ is error variance components *nrep* is the number of replicates. Restricted maximum likelihood was used to estimate the BLUP to provide a single value for each variety. Pearson’s pairwise and genetic correlations among the traits were estimated using META-R, the genetic correlation was calculated using equations from Cooper and Delacy^44^

$${\rho }_{g}=\overline{\frac{{\sigma }_{g(j{j}^{^{\prime}})}}{\overline{{\sigma }_{g\left(j\right) }}\overline{{\sigma }_{g({j}^{^{\prime}})}}}},$$

where $\overline{{\upsigma }_{g\left(j{j}^{^{\prime}}\right)}}$ is the arithmetic mean of all pairwise genotypic covariances between trait *j* and *j′* , $\overline{{\sigma }_{g\left(j\right) }}\overline{{\sigma }_{g({j}^{^{\prime}})}}$ is the arithmetic average of all pairwise geometric means among the genotypic variance components of the traits.

### Genotyping and quality control

A total of ~ 203 million 100-bp Illumina HiSeq2000 single-end reads derived from sequencing 192-plex GBS libraries were available for the 137 lines as described previously by^42^. Briefly, DNA extraction was performed using the Qiagen DNeasy 96 Plant kit, The *Ape*K1 restriction enzyme was used for library preparation as per^45^. These archived reads were processed anew to benefit from an improved SNP-calling pipeline (Fast-GBS^46^) and a more recent annotation of the soybean genome Wm82.a2.v1^47^. Genotypes were called using a minimal read depth of two reads and loci with more than 80% missing data were removed. Imputation was first performed on this catalogue of 56 K GBS-derived SNPs using BEAGLE v5^48^ to fill in any missing genotypes. We then used a set of 4.3 million SNPs derived from the whole-genome sequencing of 102 Canadian elite soybean lines^49^ as a reference panel to impute all missing loci onto the initial catalogue of GBS-derived SNPs, again using BEAGLE (Fig. S1). Among these 102 resequenced lines, 56 were in common with the association panel described above (i.e. > 40% overlap). The accuracy of imputation of such untyped loci was previously assessed^50^ and found to be 96.4%. After imputation of missing loci, VCFtools^51^ was used to retain SNPs with a minor allele frequency (MAF) ≥ 0.05 and heterozygosity ≤ 0.1, thus producing a catalogue of 2.18 M SNPs.

### Population structure

The resulting marker dataset was pruned by excluding SNPs in moderate linkage disequilibrium (LD) (r^2^ > 0.5) using PLINK^52^ to obtain a set of 14 K markers. Population structure was characterized using fastSTRUCTURE^53^ with the number of subpopulations (K) being set from 1 to 12 with 3 independent runs of each. The most likely K value was determined using a python script (“choosek”) implemented in fastSTRUCTURE based on the rate of change in LnP between successive K values. In order to better support the number of sub-populations, we used two additional tools: (i) a bootstrap consensus phylogenetic tree (2000 replicates) was constructed using the maximum likelihood method based on the Tamura-Nei model implemented in MEGA7^54^; and (ii) a principal component analysis (PCA) was performed using GAPIT^55^.

### Genome-wide association analysis

Genome-wide association between markers and the phenotypes was assessed in GAPIT and mrMLM R package v4.0 using a catalogue of 243 K SNPs and the best linear unbiased predictors (BLUP) values for each trait. Two GWAS approaches included one single-locus methods (MLM) and six multi-locus (mrMLM; FASTmrMLM, pLARmEB, ISIS EM-BLASSO, FASTmrEMMA and pKWmEB) methods were used. For significant SNP markers detection, a significant threshold, FDR ≤ 0.05 and LOD ≥ 3 were used, respectively for MLM and multi-locus methods. To account for population structure and genetic relatedness between the lines, we used the Q matrix obtained from fastSTRUCTURE and a K matrix (estimated using the EMMA algorithm^28^) as covariates. To distinct different QTL/QTN, significantly associated SNPs from the same genomic region were grouped together to form haplotype blocks, as per the LD-based method^27^, using the complete 2.18 M SNP catalogue. To be chosen as a haplotype block of interest, a block had to contain at least one peak SNP significantly associated with sulfur amino acid content. The extent of each block was used to retrieve all genes and their annotation.

### Candidate genes and their functional analysis

Only genes residing within haplotype blocks containing a peak SNP were extracted from SoyBase and were examined for their GO annotations. In order to provide more information about potential candidate genes, the electronic fluorescent pictograph (eFP) Browser (www.bar.utoronto.ca) for soybeans was used to identify in what tissues and at which developmental stages candidate genes were expressed (based on the transcriptomic data of Severin et al.^56^. We also looked for potential loss-of-function (LOF) alleles among the list of candidate genes by inspecting the catalogue of structural variants reported by Torkamaneh et al.^57^ and by examining the predicted impact of the full set of nucleotide variants located within genic regions using SnpEff ^58^. The search for such LOF alleles was limited to the 56 lines for which WGS data were available.

## Supplementary Information

Supplementary information 1.

Supplementary information 2.

Supplementary information 3.
