# Supplementary material for: Genome-wide association identifies several QTLs controlling cysteine and methionine content in soybean seed including some promising candidate genes
Source: Sci Rep. 2020 Dec 11;10:21812. doi: 10.1038/s41598-020-78907-w (PMC7733516; doi:10.1038/s41598-020-78907-w)
Supplement: Supplementary file 1 — Supplementary information 1. [file 41598_2020_78907_MOESM1_ESM.docx]

**GENOME-WIDE ASSOCIATION IDENTIFIES SEVERAL QTLS CONTROLLING CYSTEINE AND METHIONINE CONTENT IN SOYBEAN SEED INCLUDING SOME PROMISING CANDIDATE GENES.**

**Sidiki Malle^1^, Milad Eskandari^2^, Malcolm Morrison^3^, François Belzile^1*^**

^1^Département de phytologie, Faculty of Agricultural and Food Sciences and Institute for Integrative and Systems Biology (IBIS), Laval University, Quebec City, Quebec, Canada

^2^Department of Plant Agriculture, University of Guelph, Ridgetown, Ontario, Canada

### ^3^[Ottawa Research and Development Centre](http://www.agr.gc.ca/eng/?id=1180546650582), Agriculture and Agri-Food Canada, Ottawa, Ontario, Canada

**Supplementary Materials**


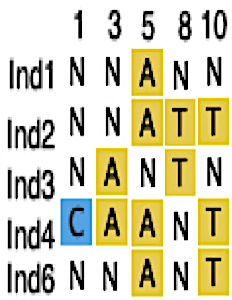


GBS raw data

BEAGLE v5


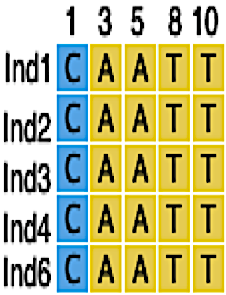


GBS imputed data

**(a)**

**(b)**


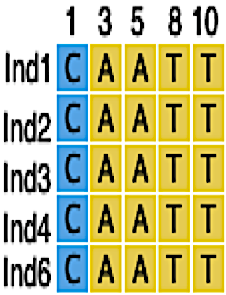


GBS imputed data


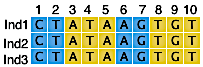


Reference haplotype (WGS)

BEAGLE v5


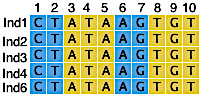


**GBS-WGS combined dataset**

Figure S1: Data integration process. a) missing genotype imputation process and b) missing loci imputation using haplotypes reference panels from WGS (Inspired from Torkamaneh et al. 2018).


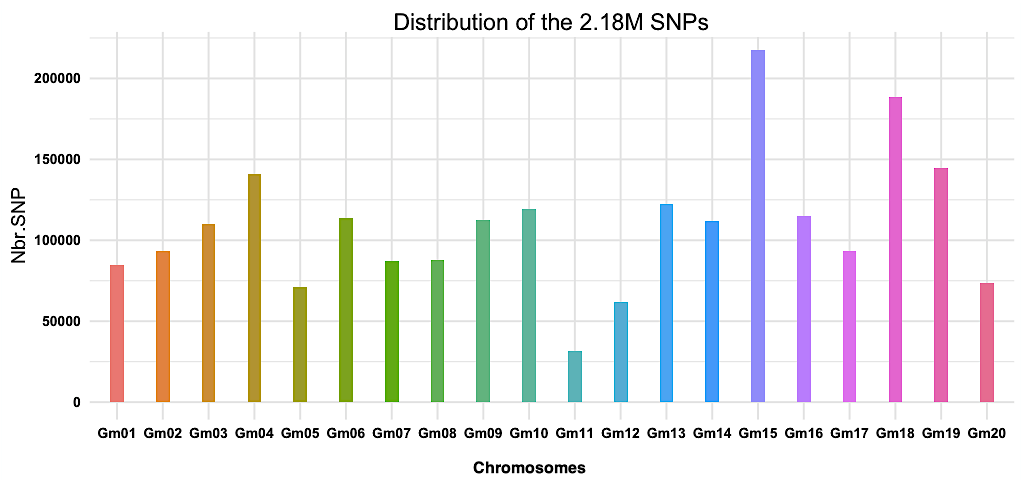


Figure S2: Distribution of the 2.18M SNPs in the soybean genome


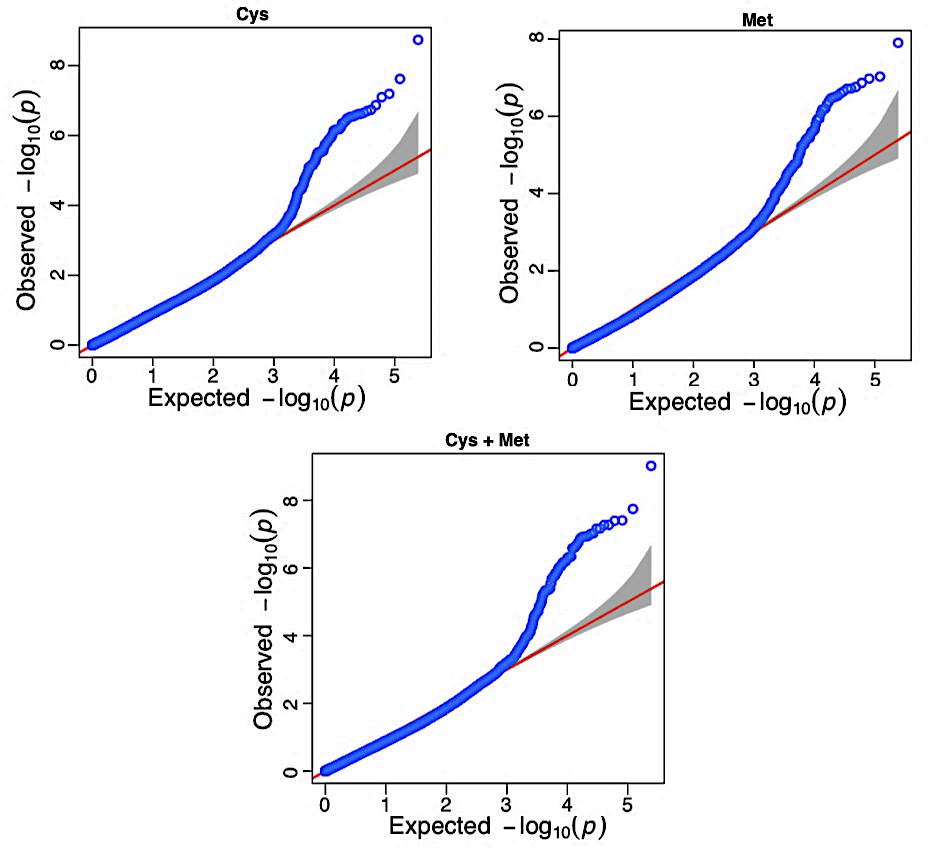


**(a)**

**(b)**

**(c)**

Figure S3: Quantile-quantile (Q-Q) plot of *p*-values for the association between SNP markers (blue dots) and Cys (a), Met (b) and the Cys + Met (c) contents in a core set of 137 Canadian soybean MG0 lines. The Y-axis is the observed negative base 10 logarithm of the p-values, and the X-axis is the expected observed negative base 10 logarithm of the p-values under the assumption that the p-values follow a uniform (0,1) distribution. The dotted lines show the 95% confidence interval for the Q-Q plot under the null hypothesis of no association between the SNP and the trait.


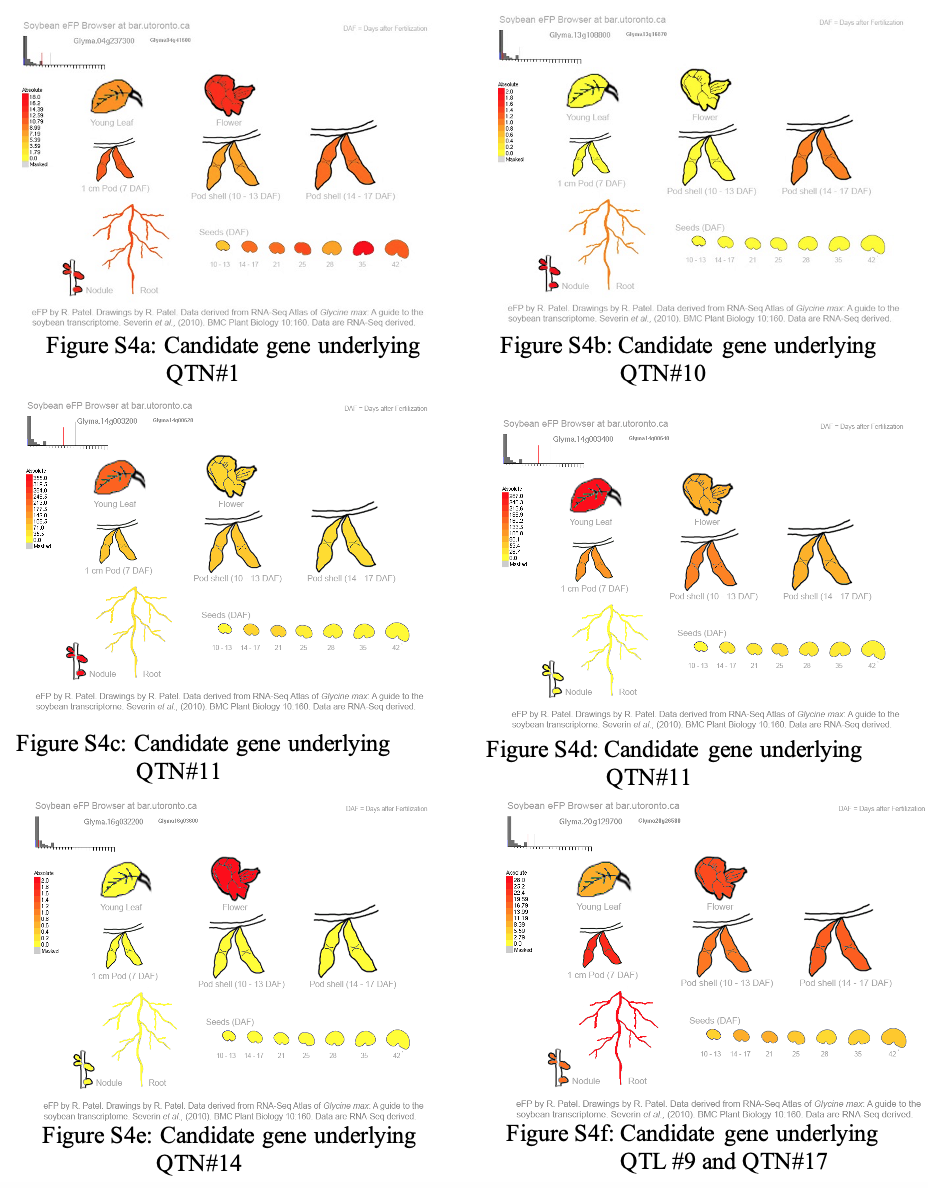


Figure S4: Expression profile of the candidate genes based on previous transcriptomic analysis in soybeans. Expression strength coded by color: yellow = low, red = high. Data derived from RNA-seq of *Glycine max*, published by Severin et al. ^1^ and download from eFP browser ^2^ ([www.bar.utoronto.ca](http://www.bar.utoronto.ca)).

1. Severin, A. J. *et al.* RNA-Seq Atlas of Glycine max: A guide to the soybean transcriptome. *BMC Plant Biology* **10**, 160 (2010).

2. Patel, R. V., Nahal, H. K., Breit, R. & Provart, N. J. BAR expressolog identification: expression profile similarity ranking of homologous genes in plant species: Expression profile similarity ranking of homologous genes. *The Plant Journal* **71**, 1038–1050 (2012).
