# Supplementary material for: Genome-wide association identifies several QTLs controlling cysteine and methionine content in soybean seed including some promising candidate genes
Source: Sci Rep. 2020 Dec 11;10:21812. doi: 10.1038/s41598-020-78907-w (PMC7733516; doi:10.1038/s41598-020-78907-w)
Supplement: Supplementary file 2 — Supplementary information 2. [file 41598_2020_78907_MOESM2_ESM.docx]

**GENOME-WIDE ASSOCIATION IDENTIFIES SEVERAL QTLS CONTROLLING CYSTEINE AND METHIONINE CONTENT IN SOYBEAN SEED INCLUDING SOME PROMISING CANDIDATE GENES.**

**Sidiki Malle^1^, Milad Eskandari^2^, Malcolm Morrison^3^, François Belzile^1*^**

^1^Département de phytologie, Faculty of Agricultural and Food Sciences and Institute for Integrative and Systems Biology (IBIS), Laval University, Quebec City, Quebec, Canada

^2^Department of Plant Agriculture, University of Guelph, Ridgetown, Ontario, Canada

^3^[Ottawa Research and Development Centre](http://www.agr.gc.ca/eng/?id=1180546650582), Agriculture and Agri-Food Canada, Ottawa, Ontario, Canada

* Correspondence: François BELZILE ([Francois.Belzile@fsaa.ulaval.ca](mailto:Francois.Belzile@fsaa.ulaval.ca)).

**Running title**: GWAS for sulfur amino acid content in soybean

**Table S1**: Genetic (bold lower diagonal) and phenotypic correlation (upper diagonal) between the different sulfur amino acid content traits.

| **Traits** | **Genetic and phenotypic correlations** | | |
| --- | --- | --- | --- |
|  | Cys | Met | Cys + Met |
| **Cys** |  | 0.81^***^ | 0.97^***^ |
| **Met** | **0.89***** |  | 0.93^***^ |
| **Cys + Met** | **0.97***** | **0.96***** |  |

## *** Significant at the 0.001 probability levels

**Table S2:** QTNs detected by at least 2/6 multi-locus methods (with a LOD score > 3.0, R^2^ ≥ 5%, only QTNs with lower LOD score are reported for overlapped QTNs between methods or traits)

| Traits | Chr | Peaks SNP (bp) | QTN  # | LOD score | R^2^ (%) | QTN  effect | Methods |
| --- | --- | --- | --- | --- | --- | --- | --- |
| Met | 4 | 50,542,407 | 1 | 4.108 | 7.82 | 0.03 | Fm/IS |
| Cys + Met | 4 | 48,478,314 | 2 | 4.238 | 5.91 | -0.07 | Fm/mr |
| Cys + Met | 6 | 46,800,853 | 3 | 5.255 | 5.82 | 0.07 | Fm/pL |
| Cys | 8 | 47,139,507^†^ | 4 | 3.697 | 5.58 | 0.07 | mr/pL |
| Cys | 8 | 43,871,472 | 5 | 4.424 | 5.65 | 0.06 | Fm/IS/mr/pL |
| Cys + Met | 10 | 3,945,266 | 6 | 4.206 | 5.65 | -0.07 | Fm/pL |
| Cys + Met | 11 | 33,737,294 | 7 | 3.072 | 5.41 | 0.11 | Fm/IS |
| Cys/Met | 12 | 11,070,552 | 8 | 4.793 | 6.65 | -0.03 | IS/mr/pL |
| Met/Cys + Met | 12 | 33,771,811 | 9 | 3.886 | 6.75 | -0.12 | Fm/mr/pL |
| Cys | 13 | 22,275,813 | 10 | 5.892 | 5.20 | 0.06 | Fm/mr/pL |
| Cys/Met/Cys + Met | 14 | 367,224 | 11 | 3.637 | 14.06 | -0.36 | Fe/Fm/IS/mr |
| Met | 14 | 6,721,937 | 12 | 3.329 | 7.05 | -0.06 | Fm/mr |
| Cys + Met | 15 | 50,494,392 | 13 | 4.261 | 13.20 | -0.12 | Fm/mr |
| Met | 16 | 3,028,024 | 14 | 5.913 | 9.01 | 0.04 | Fm/IS/pL |
| Cys | 19 | 47,167,248 | 15 | 6.234 | 6.57 | -0.13 | Fm/pL |
| Cys/Met/Cys + Met | 20 | 31,861,877^†^ | 16 | 10.669 | 25.39 | 0.31 | Fm/IS/pL |
| Cys/Met/Cys + Met | 20 | 37,049,294^†^ | 17 | 4.850 | 6.71 | 0.08 | Fm/IS/mr/pL |

† = QTLs codetected by MLM and the multi-locus methods

# = Number

R^2^ = Phenotypic variance explained by the peak SNP

Fe = FASTmrEMMA,

Fm = FASTmrMLM

IS = ISIS EM-BLASSO

Mr = mrMLM

pL= pLARmEB

**Table S3:** Degree of significance (*p*-value) of the phenotypic contrast (difference between means) for cysteine (Cys), methionine (Met) or both amino acids (Cys + Met) between lines contrasting for the allele carried at each QTL in three environments. The three trials were conducted in Ottawa (ON) in 2017 and 2018, with (I) or without (N) supplemental irrigation. Phenotypic contrasts were declared significant using a Bonferroni correction (α = 0.006 for Cys, α = 0.01 for Met and α = 0.007 for Cys + Met) and significant differences are indicated using an asterisk. When a QTL was not originally discovered as affecting Cys, Met or Cys + Met contents the phenotypic contrast was not assessed (NA).

| **QTLs** | **Trial** | **Cys** | **Met** | **Cys + Met** |
| --- | --- | --- | --- | --- |
| #1 | OT_I_2017 | 4.6E-01^ns^ | NA | NA |
|  | OT_I_2018 | 4.3E-03^*^ | NA | NA |
|  | OT_N_2018 | 7.0E-02^ns^ | NA | NA |
|  |  |  |  |  |
| #2 | OT_I_2017 | 3.7E-04^**^ | NA | 7.8E-06^***^ |
|  | OT_I_2018 | 3.0E-04^**^ | NA | 1.1E-08^***^ |
|  | OT_N_2018 | 1.3E-03^*^ | NA | 2.5E-05^***^ |
|  |  |  |  |  |
| #3 | OT_I_2017 | NA | 4.5E-04^**^ | NA |
|  | OT_I_2018 | NA | 8.5E-03^*^ | NA |
|  | OT_N_2018 | NA | 4.9E-04^**^ | NA |
|  |  |  |  |  |
| #4 | OT_I_2017 | 1.1E-04^**^ | 4.6E-04^**^ | 2.9E-07^***^ |
|  | OT_I_2018 | 3.6E-04^**^ | 2.2E-03^*^ | 9.8E-11^***^ |
|  | OT_N_2018 | 6.7E-04^**^ | 1.4E-03^*^ | 1.6E-06^***^ |
|  |  |  |  |  |
| #5 | OT_I_2017 | 5.0E-04^**^ | 1.4E-05^***^ | 1.8E-05^***^ |
|  | OT_I_2018 | 1.6E-04^**^ | 3.5E-03^*^ | 4.5E-06^***^ |
|  | OT_N_2018 | 5.5E-04^**^ | 1.5E-03^*^ | 1.1E-05^***^ |
|  |  |  |  |  |
| #6 | OT_I_2017 | 5.3E-02^ns^ | NA | 1.2E-02 ^ns^ |
|  | OT_I_2018 | 3.4E-04^**^ | NA | 2.3E-07^***^ |
|  | OT_N_2018 | 9.1E-04^**^ | NA | 2.3E-05^***^ |
|  |  |  |  |  |
| #7 | OT_I_2017 | 3.3E-05^***^ | NA | 1.6E-09^***^ |
|  | OT_I_2018 | 1.1E-04^**^ | NA | 4.7E-08^***^ |
|  | OT_N_2018 | 2.4E-04^**^ | NA | 6.1E-07^***^ |
|  |  |  |  |  |
| #8 | OT_I_2017 | 4.7E-04^**^ | 2.6E-06^***^ | 1.3E-05^***^ |
|  | OT_I_2018 | 2.5E-05 ^***^ | 3.0E-03^*^ | 2.0E-09^***^ |
|  | OT_N_2018 | 1.4E-04^**^ | 1.6E-04^**^ | 1.1E-06 |
|  |  |  |  |  |
| #9 | OT_I_2017 | 6.9E-02^ns^ | NA | 8.3E-02 ^ns^ |
|  | OT_I_2018 | 6.1E-02^ns^ | NA | 1.7E-03^*^ |
|  | OT_N_2018 | 8.2E-02^ns^ | NA | 5.3E-02 ^ns^ |

ns = not significant, ** = Significant at α ≤ 0.001, *** = Significant at α ≤ 0.0001

**Table S4:** Degree of significance (*p*-value) of the phenotypic contrast (difference between means) for cysteine (Cys), methionine (Met) or both amino acids (Cys + Met) between lines contrasting for the allele carried at each QTN in three environments. The three trials were conducted in Ottawa (ON) in 2017 and 2018, with (I) or without (N) supplemental irrigation. Phenotypic contrasts were declared significant using a Bonferroni correction (α = 0.006 for Cys, α = 0.007 for Met and α = 0.005 for Cys + Met) and significant differences are indicated using an asterisk. When a QTN was not originally discovered as affecting Cys, Met or Cys + Met contents, the phenotypic contrast was not assessed (NA). Three QTNs codetected by MLM and the multi-locus methods were excluded.

| **QTNs** | **Trial** | **Cys** | **Met** | **Cys + Met** |
| --- | --- | --- | --- | --- |
| #1 | OT_I_2017 | NA | 2.3E-02^ns^ | NA |
|  | OT_I_2018 | NA | 7.6E-02^ns^ | NA |
|  | OT_N_2018 | NA | 3.9E-01^ns^ | NA |
|  |  |  |  |  |
| #2 | OT_I_2017 | NA | NA | 3.7E-01^ns^ |
|  | OT_I_2018 | NA | NA | 7.0E-01^ns^ |
|  | OT_N_2018 | NA | NA | 3.5E-01^ns^ |
|  |  |  |  |  |
| #3 | OT_I_2017 | NA | NA | 1.3E-01^ns^ |
|  | OT_I_2018 | NA | NA | 4.8E-01^ns^ |
|  | OT_N_2018 | NA | NA | 3.8E-01^ns^ |
|  |  |  |  |  |
| #5 | OT_I_2017 | 6.6E-01^ns^ | NA | NA |
|  | OT_I_2018 | 2.2E-01^ns^ | NA | NA |
|  | OT_N_2018 | 3.0E-01^ns^ | NA | NA |
|  |  |  |  |  |
| #6 | OT_I_2017 | NA | NA | 1.2E-01^ns^ |
|  | OT_I_2018 | NA | NA | 7.3E-01^ns^ |
|  | OT_N_2018 | NA | NA | 1.6E-01^ns^ |
|  |  |  |  |  |
| #7 | OT_I_2017 | NA | NA | 3.2E-02^ns^ |
|  | OT_I_2018 | NA | NA | 1.2E-03* |
|  | OT_N_2018 | NA | NA | 1.2E-02^ns^ |
|  |  |  |  |  |
| #8 | OT_I_2017 | 4.0E-01^ns^ | 8.5E-02^ns^ | NA |
|  | OT_I_2018 | 1.0E-01^ns^ | 4.6E-01^ns^ | NA |
|  | OT_N_2018 | 6.8E-02^ns^ | 1.0E-01^ns^ | NA |
|  |  |  |  |  |
| #9 | OT_I_2017 | NA | 3.1E-01^ns^ | 9.2E-01^ns^ |
|  | OT_I_2018 | NA | 1.7E-01^ns^ | 1.2E-01^ns^ |
|  | OT_N_2018 | NA | 3.5E-01^ns^ | 1.8E-01^ns^ |
|  |  |  |  |  |
| #10 | OT_I_2017 | 7.6E-01^ns^ | NA | NA |
|  | OT_I_2018 | 9.0E-01^ns^ | NA | NA |
|  | OT_N_2018 | 8.0E-01^ns^ | NA | NA |
| #11 | OT_I_2017 | 1.1E-01^ns^ | 3.4E-01^ns^ | 1.3E-01^ns^ |
|  | OT_I_2018 | 8.8E-02^ns^ | 1.8E-02^ns^ | 3.4E-02^ns^ |
|  | OT_N_2018 | 3.2E-01^ns^ | 4.3E-01^ns^ | 3.2E-01^ns^ |
| #12 | OT_I_2017 | NA | 3.3E-01^ns^ | NA |
|  | OT_I_2018 | NA | 8.0E-02^ns^ | NA |
|  | OT_N_2018 | NA | 2.1E-02^ns^ | NA |
| #13 | OT_I_2017 | NA | NA | 1.3E-01^ns^ |
|  | OT_I_2018 | NA | NA | 8.4E-01^ns^ |
|  | OT_N_2018 | NA | NA | 7.2E-02^ns^ |
| #14 | OT_I_2017 | NA | 4.0E-02^ns^ | NA |
|  | OT_I_2018 | NA | 5.8E-01^ns^ | NA |
|  | OT_N_2018 | NA | 3.4E-01^ns^ | NA |
| #15 | OT_I_2017 | 9.3E-01^ns^ | NA | NA |
|  | OT_I_2018 | 4.1E-01^ns^ | NA | NA |
|  | OT_N_2018 | 2.4E-01^ns^ | NA | NA |

ns = not significant, * = Significant at α ≤ 0.005.
